# Nanoparticles in the Food Industry and Their Impact on Human Gut Microbiome and Diseases

**DOI:** 10.3390/ijms22041942

**Published:** 2021-02-16

**Authors:** Merry Ghebretatios, Sabrina Schaly, Satya Prakash

**Affiliations:** Biomedical Technology and Cell Therapy Research Laboratory, Department of Biomedical Engineering, Faculty of Medicine, McGill University, 3775 University Street, Montreal, QC H3A 2B4, Canada; merry.ghebretatios@mail.mcgill.ca (M.G.); sabrina.schaly@mail.mcgill.ca (S.S.)

**Keywords:** gut microbiome, microbiome, gut model, nanoparticles, nanotoxicity, probiotics, dysbiosis, silver, iron oxide, zinc oxide, titanium dioxide, silicon dioxide, food nanotechnology, gut health

## Abstract

The use of inorganic nanoparticles (NPs) has expanded into various industries including food manufacturing, agriculture, cosmetics, and construction. This has allowed NPs access to the human gastrointestinal tract, yet little is known about how they may impact human health. As the gut microbiome continues to be increasingly implicated in various diseases of unknown etiology, researchers have begun studying the potentially toxic effects of these NPs on the gut microbiome. Unfortunately, conflicting results have limited researcher’s ability to evaluate the true impact of NPs on the gut microbiome in relation to health. This review focuses on the impact of five inorganic NPs (silver, iron oxide, zinc oxide, titanium dioxide, and silicon dioxide) on the gut microbiome and gastrointestinal tract with consideration for various methodological differences within the literature. This is important as NP-induced changes to the gut could lead to various gut-related diseases. These include irritable bowel syndrome (IBS), inflammatory bowel disease (IBD), celiac disease, and colorectal cancer. Research in this area is necessary as the use of NPs in various industries continues to grow along with the number of people suffering from chronic gastrointestinal diseases.

## 1. Introduction

**Figure 1:**
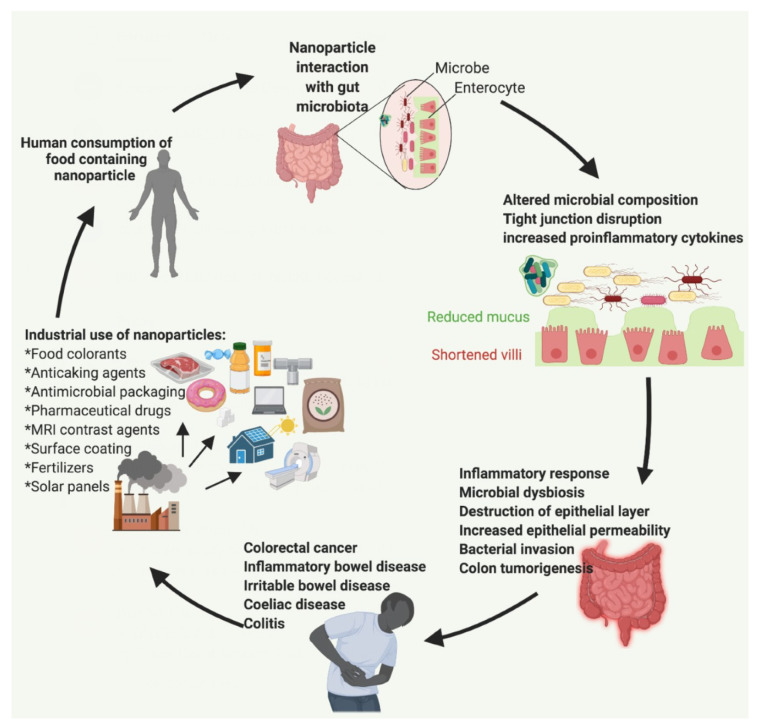
Contamination due to industrial use of nanoparticles in food processing and other industries and their potential impact on human health and diseases through the gut microbiota. Created with BioRender.com.

This review presents a discussion of various papers on the impact of inorganic nanoparticles (NPs) on the gut microbiome and the gastrointestinal tract (GIT) in relation to health. The NPs discussed include silver (Ag NPs), silicon dioxide or silica (SiO_2_ NPs), titanium dioxide (TiO_2_ NPs), iron oxide (Fe_2_O_3_ NPs), and zinc oxide (ZnO NPs). The vast and increasing industrial applications of NPs are outlined with a focus on the food industry as this has provided NPs direct access to the GIT and gut microbiome. Differences between studies in terms of NP characteristics, study design, and techniques are highlighted to provide a basis for the conflicting findings in the literature. Finally, NP-induced changes to microbial composition are related with disease-associated alterations of the gut microbiome. Ultimately, NP consumption through food can alter the composition of the gut microbiota and the GIT towards disease-related states, potentially promoting pathogenesis and contributing to various autoimmune and gut-related diseases (Figure 1).

## 2. Human Exposure to Food Nanoparticles and Their Industrial Applications

Human exposure to NPs, clusters of atoms ranging from 1 to 100 nm in one dimension, is nearly unavoidable [1]. As of March 2015, there were 1814 products (representing 622 companies and 32 countries) containing nanomaterials and 117 of them fit under the “food and beverage” category [2]. Of these 117 products, 47% were advertised as having at least one nanomaterial while 62 of these products were composed of more than one nanomaterial. The products were grouped into five categories based on nanomaterial type and 37% fit under “metals” and “metal oxide nanoparticles”. TiO_2_, SiO_2_, and ZnO were the most produced nanomaterials by mass, worldwide, and Ag NPs made up 24% of the most popularly advertised nanomaterials. It is estimated that children consume 1.6–3.5 ug/kg body weight per day (bw/day) and adults consume 1.3–2.7 μg/kg bw/day of Ag NPs [1]. Humans consume an estimated 1.8 mg/kg bw/day of SiO_2_ NPs from food [3]. E171 food additives containing up to 43% TiO_2_ NPs have been used since 1969 in many food products, including chewing gum [4]. It is estimated that exposure to TiO_2_ NPs is 0.2–0.4 mg/kg bw for infants and the elderly, and 5.5–10.4 mg/kg bw for children [4]. Other studies suggest intake may be much higher due to the increased use of NPs in a variety of industries in addition to the food industry [2]. 

The nano-scale size of NPs affords them unique physicochemical properties that make them suitable for applications in various industries [5] (Table 1). Inorganic NPs are widely used for processing, packaging, and nutrition [6]. TiO_2_ has been used as a coloring agent and enhancer for dairy products, beverages, seeds, processed foods, toothpaste, and even medications [7]. It is also used in coating candies [1]. SiO_2_ is registered within the EU as a food additive (E551) for maintaining flow in powder products and carrying flavors in food [6]. Ag NPs are used in food packaging as they have diverse antibacterial properties [8]. These antimicrobial NPs enter bacterial cells and interfere with respiration, phosphate uptake, DNA replication, and protein modifications [9]. Finally, ZnO NPs are used in antimicrobial food packaging and supplements among other applications [10,11].

NPs also have vast applications in fields outside of the food industry. For example, 12% of cosmetic products are advertised to have Ag and TiO_2_ NPs [2]. Similarly, Ag NPs are used as coatings for computer keyboards to protect against microbes [2]. Such wide-ranging applications of nanomaterials in various industries, as summarized in Table 1, allows for greater human exposure. This widespread application of nanotechnology likely increases direct human ingestion of these NPs providing them better access to the host gut microbiota through the GIT [6]. Indeed, research has shown that Ag NPs accumulate in the stomach, duodenum, ileum, jejunum, and colon [6]. TiO_2_ NPs were also found stored in the stomach and colon, while SiO_2_ NPs have been shown to be distributed in the stomach, ileum, and colon. This has caused understandable concern over the potential negative impact of NPs on the gut microbiome and the resulting effect on human health. 

## 3. Nanoparticles as Bioactive Agents

To be able to assess the risk associated with exposure to NPs, their stability in the GI lumen and their method of absorption must be well understood. Data shows that the stability, aggregation, and surface properties of NPs can change depending on their interactions with the GIT [31]. For instance, physical forces (peristalsis), osmotic concentration, pH, digestive enzymes, the presence of other foods, endogenous biochemicals, and commensal microbes may have an impact on NP characteristics. In turn, any changes to the NP will influence its absorption and how it may affect the gut microbiome. Cellular uptake of NPs may be endocytosis-dependent or endocytosis-independent [5]. Researchers suggest that the mechanism is influenced by the presence of microvilli as endocytosis of NPs is reduced in cells with extensive microvilli [5]. NPs were shown to pass between epithelial cells of the GIT by paracellular transport which involves disrupting the tight junctions that hold epithelial cells together. In other cases, goblet and M-cells can readily take in NPs through endocytosis. One study assessed the impact of NP size and agglomeration state on the levels and mechanisms of NP internalization [32]. It was found that well-dispersed silica NPs entered cells by Caveolae-mediated endocytosis whereas an increase in the agglomeration state caused a shift towards NP uptake via micropinocytosis. After NPs enter cells, studies have shown they can escape the lysosomal or endosomal compartment and spill into the cytosol. In this way, NPs have been shown to impact the mucus layer, mucus-producing cells, and intestinal epithelial cells [5]. These barriers serve to protect the host from pathogens, among other things, and their disruption can lead to autoantigen exposure and aberrant damage to cells [5]. Such impact of NPs on cells can produce danger signals, which further disrupts barrier function and threatens gut dysbiosis [5]. Therefore, NPs can negatively impact gut barrier function, potentially leading to the disruption of microbial homeostasis.

## 4. Role of the Gut Microbiota and the Impact of Dysbiosis

The human GIT houses trillions of microorganisms such as bacteria, viruses, and fungi [1]. This microbiota plays an important role in GI physiology including gastric secretion, gut motility, mucosal permeability, mucosal blood flow, and more [1]. The microbiota also aids in the absorption of nutrients from foods that are indigestible to the human body [1]. In a healthy adult, 80% of fecal microbiota can be classified into three dominant phyla; Bacteroidetes, Firmicutes, and Actinobacteria [33]. Environmental factors such as diet, toxins, drugs, and pathogens have the potential to alter the gut microbiota [34]. Research has shown that dysbiosis is associated with the development of inflammatory bowel disease (IBD), irritable bowel syndrome (IBS), and metabolic syndrome [35]. Moreover, the ratio of Firmicutes/Bacteroidetes (F/B) is particularly indicative of the overall health of the GIT. For example, an elevated F/B ratio is associated with obesity, while a decrease in F/B is directly related to weight loss. Due to the health consequences of altering the microbiome, and the vast human exposure to NPs, more research must be done to investigate NPs in the food industry and their effects on the gut microbiota in relation to various diseases (Figure 1).

### 4.1. Dysbiosis Is Associated with Irritable Bowel Syndrome and Inflammatory Bowel Disease

The most common gastrointestinal disorders are IBD and IBS [36]. Chron’s disease (CD) and ulcerative colitis (UC) are the most prevalent forms of IBD and involve chronic relapsing inflammation of the intestinal mucosa [36]. IBS pathogenesis is poorly understood with no known physical causes and is, therefore, characterized by symptoms such as abdominal pain, diarrhea, and constipation [37]. In both cases, the gut microbiome is thought to play a role in the pathogenesis [38]. One study analyzed 1792 participant’s stool samples and compared them to that of 1025 control participants [36]. The study found that IBD samples had microbial dysbiosis [36]. Overall, IBD patients have decreased functional diversity, reduced microbial stability, lower populations of Firmicutes, increased Bacteroidetes and increased *Enterobacteriaceae* [39]. IBS is also associated with changes to the microbiota. For example, one study analyzed intestinal samples of individuals with IBS and found them to have reduced aerobic bacteria compared to healthy controls [40]. For instance, IBS samples show an increase in Actinomyces, Streptococcus, and *Blautia* genera [36]. Other studies have also associated IBS and colitis in mice with increased genus Alistipes, *Bacteroides,* and *Prevotella,* and increased phylum Bacteroidetes [6].

### 4.2. Dysbiosis Is Associated with Colorectal Cancer and Celiac Disease

Celiac disease and colorectal cancer are also associated with reduced microbial diversity and richness compared to healthy control subjects [41]. Colorectal cancer (CRC) has been associated with increased levels of Bacteroidetes and Proteobacteria and lower levels of Firmicutes [41]. However, more research must be done to identify what microbes aid tumorigenesis [41]. There is also evidence for microbiota-dependent colon tumorigenesis. This process begins with goblet cell loss and subsequent bacterial invasion into epithelial crypts leading to tumor formation [41,42]. Indeed, microbial dysbiosis was shown to promote tumor formation in germ-free mice [42]. Celiac disease is an intestinal inflammatory disorder caused by an autoimmune response to dietary gluten. The disease is also associated with an increase in epithelial permeability which allows luminal antigens into the submucosa [43]. One study characterized the bacterial composition of celiac disease patients finding lower levels of IgA-coated fecal bacteria [43]. The ratio of gram-positive to gram-negative bacteria was also lower in these patients. Celiac disease samples showed reduced levels of Bifidobacterium, *Clostridium histolyticum*, *C. lituseburense*, and *Faecalibacterium prausnitzii* as well as increased levels of the *Bacteroides-Prevotella*. The IgA coating on the *Bacteroides-Prevotella* group was also reduced in celiac patients.

An important consideration is that the presented data are mostly correlational making it possible for the microbial alterations to be a result of disease rather than a cause. However, this has been addressed with experimental studies showing dysbiosis occurring before disease onset. For example, children with the haplotype for celiac disease showed altered microbiomes prior to disease progression [44]. Additionally, celiac disease is a result of immune overreactions to gluten, so it is plausible that the presence of bacteria capable of digesting gliadin would neutralize autoimmunity and, therefore, help the condition while others may worsen it [34].

## 5. Impact of Inorganic Nanoparticles on the Gut Microbiota

### 5.1. Ag NPs

A group of researchers used Citrate-stabilized Ag NPs (10, 75, 110 nm) on male and female Sprague-Dawley rats [45]. The NPs were administrated via oral gavage twice daily for 13 weeks at three different doses (9, 18, 36 mg/kg bw/day). The NPs showed sex, size, and dose-dependent antimicrobial effects on the microbial population in the mucosa as determined by culturing ileal tissues. In short, all NP sizes had antimicrobial effects but not at all doses and this depended on the sex of the animal. They observed decreased Firmicutes phyla in the 10 nm Ag NP group at most doses in males and females. Bacteroidetes increased at the lower dose of 110 nm Ag NPs in females but decreased in males. At a high dose of 110 nm Ag NP, Firmicutes decreased and Bacteroidetes increased in both male and female rats. Researchers observed a decrease in the genus *Lactobacillus* (10, 75 nm). Bacteroidetes increased in male and female rats given higher doses of 110nm Ag NP and Bifidobacterium increased in male rats given the lowest dose of 75 and 110 nm Ag NP. Comparatively, in a study using 12 nm, 2.5 mg/kg bw/day Ag NPs on male mice for 7 days, 16S rRNA sequencing showed a reduction in the F/B ratio and body weight loss was observed [6]. The genera *Alistipes*, *Bacteroides,* and *Prevotella* increased, while *Lactobacillus* decreased.

Other studies have also discovered Ag NP-induced changes in the gut microbiome using different techniques. For example, a bacterial community established from a healthy donor’s stool was exposed to 10 nm Ag NPs (0–200 mg/L) for 48 h resulting in 20% reduced gas production by the microbes at high doses (100 and 200 mg/L) [46]. Reduced gas production was attributed to CO_2_, indicating that Ag NPs may have reduced the metabolic activity of microbes in the sample. Furthermore, there was a shift in the community structure as seen via analysis of PCR-denaturing gradient gel electrophoresis (DGGE) profiles. For instance, there was a 26–36% difference in banding profiles at increasing Ag NP concentrations. DNA from the treated samples also underwent 16S rRNA sequencing which showed 57% reduction of the gram-negative anaerobe, *Bacteroides ovatus,* among other alterations. Authors hypothesized that the thinner cell membranes of gram-negative bacteria are what makes them more vulnerable to toxicity. Of interest is that *Escherichia coli* also increased (50–80%) after Ag NP treatment of the sample. This study found that Ag NP effects were different from that of ionic silver, indicating the impact of size. Ag NP treated groups also showed strikingly different fatty acid signatures (i.e., capric fatty acids were reduced by 80–96%) compared to controls receiving no treatment. This study concluded that Ag NPs impact the intestinal microbiota at certain concentrations.

Researchers have also orally treated male Sprague Dawley rats with polyvinylpyrrolidone (PVP) coated cube and sphere-shaped Ag NPs (45 and 50 nm) at a dose of 3.6 mg/kg bw/day for 14 days [47]. *Clostridium* spp., *Bacteroides uniformis*, *Christensenellaceae*, and *Coprococcus eutactus* were reduced in rats treated with cube-shaped Ag NP. *Oscillospira* spp., *Dehalobacterium* spp., *Peptococcaeceae*, *Corynebacterium* spp., and *Aggregatibacter pneumotropica* were reduced in rats treated with sphere-shaped Ag NPs. In another study, female mice were orally exposed to food pellets containing 55 nm PVP-coated Ag NPs [9]. This was done for 28 days using three doses (11.4, 114, 1140 ug/kg bw/day). Upon analysis of fecal microbiota, researchers found a dose-dependent increase in the F/B ratio, *Coprococcus*, *Lactobacillus*, and *Blautia*, as well as a decrease in *Bacteroides* and *Mucispirillum*.

By contrast, other studies have reported that Ag NPs have little to no impact on the gut microbiome. For instance, one group studied cecal microbiota using Ag NPs (20 nm and 110 nm) coated in either PVP or citrate [48]. Male mice received 10 mg/kg bw/day via oral gavage for 28 days. This study concluded that there are no changes in the relative abundance of any phylum, microbial community structure, or microbial diversity regardless of the size and coating of the Ag NPs administered. While these results contradict the previously discussed studies, they are influential as the researchers showed that their positive control group (animals dosed with the antibiotic cefoperazone) had significantly reduced levels of Bacteroidetes, increased Firmicutes, and reduced diversity. Another study exposed female Wistar rats to 14nm PVP-stabilized Ag NPs via gavage (2.25, 4.5, 9 mg/kg bw/day) [49]. The study compared this to the administration of different doses (9 mg/kg bw/day) of Ag-acetate for 28 days. Cecal amounts of Firmicutes and Bacteroidetes were unchanged by either form of sliver. In a study using in-vitro batch fermentation models inoculated with human fecal matter, treatment with 1 ug/mL Ag NPs increased the F/B ratio [50]. Ag NPs also induced functional differences in cell motility, translation, transport, and xenobiotics degradation. Overall, however, it was concluded that Ag NPs did not alter the composition of the core microflora or their short-chain fatty acid (SCFA) profiles. Furthermore, one study synthesized and administered two aqueous suspensions of Ag NPs (NP1 and NP2 daily at 500 mg/dm^3^) to mouse models of ulcerative colitis and Crohn’s disease [51]. This improved colitis in the mouse models of inflammation. While the NPs did not alter the total number of bacteria, NP1 non-significantly reduced the number of Lactobacillus sp., and increased the number of Clostridium perfringens and *E. coli* in the mouse stool. By contrast, NP2 increased the number of Lactobacillus sp. and decreased the number of C. perfringens and *E. coli*. Therefore, some changes to the gut microbiome induced by Ag NPs can contribute positively to the health of the host. Overall, many studies show that Ag NPs have an impact on the gut microbiome while others suggest there is no impact. 

### 5.2. ZnO NPs

One study exposed hens to 25 mg/kg, 50 mg/kg, and 100 mg/kg of ZnO NPs and sequenced the ileal microbiota [52]. They found that bacterial community richness was reduced as the dose increased. Specifically, the abundance of Firmicutes and *Lactobacillus* showed a negative correlation with NP exposure. *Lactobacillus* is the predominant bacteria in animal and human ilea, therefore, its reduction caused by ZnO NPs is problematic. There were also increased populations of Bacteroidetes, Fusobacteria, and Bacilli. In a study comparing the effects of 600 mg Zn/kg (Nano-ZnO) and 2000 mg Zn/kg (ZnO) on piglets, 16S rRNA analysis showed that bacterial richness and diversity increased in the ileum but decreased in the cecum and colon [53]. In the ilium, researchers found higher levels of Streptococcus along with reductions in *Lactobacillus*. In the colon, however, *Lactobacillus* increased while *Oscillospira* and *Prevotella* decreased. Another study used human microbiota from healthy donors and found that ZnO NPs inhibited SCFA production [54]. Taken together, the small amount of data suggest that ZnO NPs can cause changes to the composition of the intestinal microbiota, such as a reduction in genus *Lactobacillus,* while also altering their metabolic activity as seen by changes in SCFA production.

### 5.3. Fe_2_O_3_ NPs

There are few studies on the impact of iron (Fe_2_O_3_) NPs on the gut microbiome yet the available data suggest these NPs are safe and non-toxic. It has been shown that ferrous sulfate-supplemented diets promote reduced Bifidobacterium and increased sulfate-reducing bacteria (Desulfovibrio) which are both unfavorable outcomes [55]. However, nano Fe(III) was found to be a better alternative having no negative impact on the gut microbiome. Additionally, the authors discovered that the use of iron NPs resulted in less iron exposure to the microbiota when compared to iron salt. This means that the nano form has limited solubility in the gut, allowing for potentially reduced contact with the microbiome. Similarly, another study noted that iron NPs increased the diversity and health of the microbiota marked by an increase in Lactobacillus [56]. 

### 5.4. SiO_2_ NPs

The impact of SiO_2_ NPs on the gut microbiome has not been studied extensively. However, one study has shown SiO_2_ NPs to have some negative impact on the gut microbiota. Researchers exposed mice to human-relevant doses of SiO_2_ NPs (2.5 mg/kg bw/d) for one week and found increased diversity and richness of the microbial community [6]. There was an increase in Firmicutes and Proteobacteria as well as reduced Bacteroidetes and *Lactobacillus*. Of note, the rate of absorption of precipitated or fumed (amorphous) silicate allows it to accumulate in the gut lumen, providing more time for toxic effects on the gut microbiome. This means fumed silicate may have the potential to do more harm.

### 5.5. TiO_2_ NPs

TiO_2_ NPs seem to have less pronounced effects on microbial composition according to some studies. One study used a defined model intestinal bacterial community to test the toxicity of food-grade TiO_2_ NPs at a dose relevant to the amount present in the human intestines after having 1–2 pieces of gum or candy [57]. The study found only minor reductions in *Bacteroides ovatus* and an increase in *Clostridium cocleatum,* concluding that TiO_2_ NPs have no major impact on the gut microbiota at low concentrations. However, it is important to recognize that chronic exposure is not represented in this study as it was completed after only 48 h of NP exposure. Nevertheless, another study exposing mice to 2.5 mg/kg bw/day of TiO_2_ NPs for 7 days also found no changes to the composition of fecal microbiota [6]. Similarly, a study using an in-vitro Human Gut Simulator system to assess the impact of TiO_2_ NPs on the gut microbiome found community density was reduced but there was no impact on diversity and evenness, or microbial functionality and fermentation [58]. These studies demonstrate that TiO_2_ NPs have little impact on the gut microbiome.

By contrast, other studies have shown TiO_2_ NPs can drastically alter the gut microbiota. One study, using a model microbial community inside a model colon, administered 3 mg/L TiO_2_ for 5 days and found alterations in the microbial community’s phenotype [54]. Specifically, there were significant changes to the bacterial metabolites produced, including SCFAs. In another study, the rutile form of TiO_2_ NPs caused increased proteobacteria while the anatase form did not, and the genus *Prevotella* decreased significantly in both cases [59]. Additionally, rutile NPs increased *Rhodococcus* while anatase NPs increased *Bacteroides*. Furthermore, one study assessed the effects of both food grade (i.e., commercially available) and industrial-grade TiO_2_ NPs [60]. Food-grade TiO_2_ NPs most inhibited an expected shift from Proteobacteria to Firmicutes. Food-grade NPs also reduced the pH of the colon more than industrial-grade NPs did. Findings in this study show that the chemical and physical properties of TiO_2_ NPs influence the resulting changes in the microbiome. Another study assessed the oral toxicity of TiO_2_ NPs at different doses (0.16, 0.4, 1 g/kg) in Wistar rats [61]. Treated rats had a slight injury to the heart and liver which authors attribute to disturbances in energy and amino acid metabolism as well as the gut microflora environment. In a study testing TiO_2_ NP hepatotoxicity, rats were administered 29 nm TiO_2_ NPs at various doses (0, 2, 10, 50 mg/kg) each day for 90 days [62]. Sequencing showed that microbial diversity increased dose-dependently. There was an increase in *Lactobacillus_reuteri* and a decrease in *Romboutsia* in the rat’s feces. This change in the microbiota led to an increase in the production of Lipopolysaccharides (LPS). Overall, the impact of TiO_2_ NPs on the gut microbiota is controversial. 

As discussed, many studies have researched inorganic NPs to determine their impact on the gut microbiota. Unfortunately, the findings conflict with one another making it difficult to determine the precise impact of these NPs on the gut microbiota. The dose, size, coating, and shape of the NPs used differ greatly between studies and may account for the variability in findings. Additionally, the gut model used for the study, the mode of administration of NPs, the origin of analyzed samples, and the method of analysis are different between studies, which may have further contributed to the conflicting data. Table 2 summarizes the physicochemical properties of NPs, experimental designs, and techniques used in each study along with the results obtained.

## 6. Impact of Inorganic Food Nanoparticles on the Gastrointestinal Tract

The small intestine mucosal layer is thinner than that of the stomach and is less attached to the epithelial layer [31]. This is to allow for absorption of nutrients into cells, however, the mucus is still needed to trap and immobilize larger unwanted particles including bacteria [63]. The outermost layer of the GIT epithelial layer contains villi and microvilli, which point towards the lumen to increase the surface area and enhance absorption [31]. The epithelial layer is made up of specialized cells like goblet cells, responsible for secreting mucus, as well as M-cells, which transport material from the lumen across the epithelial barrier. These cells, among others, play a part in maintaining gut homeostasis [31]. Because of this delicate balance, any impact NPs may have on villus structure or mucus secretion will affect the gut’s natural interaction with the microbiome. This has the potential to disrupt homeostasis possibly leading to pathology. Here, some of the effects of food NPs on the GIT are discussed.

### 6.1. Ag NPs

In one study, Ag NPs did not affect the expression of the mucin gene *MUC2*, however, there was a decrease in *MUC3* expression in the ileum that was most prominent in female rats [45]. The *MUC* genes encode members of the gel-forming mucin protein family which are secreted into the mucus layer [64]. The genes for microbial recognition called toll-like receptors (TLR2/4) and *NOD2* were downregulated depending on the dose given and sex of the rats [45]. Furthermore, the expression of T-cell regulatory genes (*FOXP3, GPR43, IL-10, TGF-β*) decreased, particularly at low and medium doses. The authors also noted that the observed changes in genetic expression seemed to depend more on Ag NP interactions based on their dose and size rather than their release of ions. Another study administered a smaller size and dose of Ag NP (12 nm, 2.5 mg/kg bw/day) to male mice via oral gavage for 7 days [6]. Ag NP treated mice exhibited colitis-like symptoms such as increased disease activity index, histological scores, intestinal epithelial microvilli, tight junction disruption, and increased pro-inflammatory cytokines.

In contrast, a study using histological analysis did not find any intestinal damage or structural alterations in ileal villi, goblet cells, or the glycocalyx across all groups treated with Ag NPs [9]. Similarly, another study showed that Ag NP (14nm) treated rats did not experience toxicological effects [49]. Furthermore, a previously mentioned study showed that Ag NPs can positively impact the GI tract [51]. The researchers first achieved reproducible colitis in mice, as evidenced by increased macro- and microscopic damage scores, by orally administering dextran sulfate sodium (DSS). Administration of Ag NP2 (500 mg/dm^3^, 100 μL/animal, once daily) significantly decreased the total macroscopic score, effectively attenuating DSS-induced colitis. Ag NP1 (500 mg/dm^3^, 100 μL/animal, intracolonic, once daily) non-significantly decreased the macroscopic score but still significantly reduced the colon damage score. Additionally, microscopic damage (i.e. loss of mucosal architecture, presence of crypt abscesses, and extensive cellular infiltration) observed in DSS-treated mice was alleviated after treatment with NPs. The study even showed that NPs alleviated colonic injury in a mouse model mimicking CD. Thus, like their impact on the gut microbiota, Ag NPs have been shown to both positively and negatively impact the GIT. 

### 6.2. SiO_2_ NPs

SiO_2_ NP-treated mice exhibited significant increases in pro-inflammatory cytokines in the small bowel and colon [6]. This was confirmed by hematoxylin and eosin (H & E) staining after NP ingestion which revealed severe destruction of the epithelial layer and loss of crypts in colon segments. Another study found toxic effects unrelated to the GIT after orally exposing rats to 100, 1000, or 25,000 mg/kg bw/day of synthetic amorphous silica (SAS) [65]. SAS is used in food and drugs and contains up to 43% nano silica between 5 and 200 nm in size. Elevated tissue silica levels were observed in the spleen after 84 days of exposure to the highest dose of SAS. Moreover, after 84 days, liver fibrosis was observed, indicating potential long-term effects. 

By contrast, other studies have found silica to have no effect on the gut. Colloidal silica particles, differing in size (20 nm and 100 nm), were orally administered to Sprague-Dawley rats [66]. A ninety-day repeated dose (2000 mg/kg, 1000 mg/kg or 500 mg/kg) study was conducted. There were no clinical changes, toxic effects, or histopathological findings in any of the rat groups. Similarly, researchers orally administered 2.5 mg/day of amorphous silica NPs to mice, for 28 days, of different diameters and surface properties (70, 300, and 1000 nm) [67]. The three NPs were absorbed, in the intestine, to different degrees, indicating that particle diameter and surface properties are determinants. Moreover, after 28 days, there was no significant difference in hematological, histopathological, and biochemical properties in the control mice and mice given silica. This study thus suggests that silica NPs are safe for food production. Overall, some studies show that silica NPs negatively impact the GIT while others show there is no such impact. 

### 6.3. TiO_2_ NPs

One study showed that rutile NPs (a crystalline phase of TiO_2_ NPs) increased the length of intestinal villi and caused irregular arrangement of epithelial cells [59]. Of note is that this study used human exposure relevant doses on mice for 28 days. The findings were more pronounced with the use of rutile NPs as compared to anatase NPs. In another study, rats were given 10 mg/kg bw/day of food-grade TiO_2_, an approved white pigment in Europe, orally for 7 days [68]. Intestinal inflammation, preneoplastic lesions, and growth of aberrant crypt foci arose 100 days after treatment, indicating an increased risk for T helper 17-driven autoimmune diseases and colorectal cancer. In this way, NPs can negatively impact the health of individuals that are chronically exposed. These results are concerning since the use of TiO_2_ NPs is very popular in candies which are mostly consumed by children.

### 6.4. Fe_2_O_3_ NPs

One study investigated iron oxide NPs in food and how their consumption impacts gut morphology in the *Bombyx mori* silkworm [69]. *B. mori* were fed 0.3%, 1.5%, and 3% by weight of the iron oxide NPs and fixed with staining for analysis. Results showed morphological changes in the gut including increased amounts of goblet cells for the 1.5% treatment group. In those fed 1.5% NPs, there was pseudostratified epithelium in the gut lining and a loss of goblet cells. Finally, in those treated with 3% NPs, epithelial cells were irregularly distributed and there was apoptosis resulting in increased intracellular space.

Another study had different findings. Researchers assessed both iron oxide and SiO_2_ NPs in Sprague-Dawley rats. One group was orally administered 244.9, 489.8, and 979.5 mg/kg SiO_2_ NPs that were 12nm and spherical in shape [70]. Another group received 1030.5 mg/kg Ag NPs and 1000 mg/kg Fe_2_O_3_ NPs. In this 13-week repeated toxicity study, the SiO_2_ and iron oxide NPs were not associated with systemic toxicity or any changes in hematological, serum biochemical, or histopathological lesions. However, the same study showed that Ag NPs increased serum alkaline phosphatase, calcium, and lymphocyte infiltration in the liver and kidney. This indicates a potential for silver NPs to cause systemic toxicity due to its systemic distribution but not SiO_2_ or Fe_2_O_3_ NPs. The toxicity assessments were done according to the Organization for Economic Cooperation and Development (OECD) test guideline 408. 

### 6.5. ZnO NPs

One study compared the effects of 600 mg/kg ZnO NPs and 2000 mg/kg ZnO on piglets for 14 days [53]. Antioxidant enzyme (Cu-Zn superoxide dismutase, glutathione peroxidase) and tight junction protein mRNA expression (zonula occludens protein-1, and occluding) increased in both nano and traditional ZnO treatment groups compared to controls. However, the ZnO NP treatment group had lower expression than the traditional group. Thus, the effect of weaning stress on piglets seems to be better alleviated by traditional ZnO than by the lower dose of Nano-ZnO. The mRNA expression of cyclin-dependent kinase-4 (CDK-4) increased and Caspase3 decreased in both groups compared to controls. However, Nano-ZnO had lower CDK-4 expression compared to the traditionally treated group. CDK4 is a marker for proliferation and Caspases are proteins involved in apoptosis meaning that both nano and traditional ZnO treatments promote proliferation and inhibit apoptosis in enterocytes. Jejunal villus height and the ratio of villus height to crypt depth were unchanged in the ZnO NP treatment group compared to controls. However, this increased significantly in the traditionally fed group compared to the controls. Crypt depth did not change across all groups. Taken together, this suggested that ZnO NPs can improve the morphology of the jejunum just as traditional high doses of ZnO can. In another study, researchers green synthesized ZnO NPs from *Paeonia tenuifolia* root extract and showed them to have antioxidant and anti-inflammatory effects [71]. Therefore, the use of green synthesized ZnO NPs may mitigate potential harms.

## 7. Discussion

### 7.1. Overall Impact of Inorganic NPs on the Gut Microbiome and Intestinal Tract

According to the studies discussed, four inorganic NPs can alter the gut microbiota which excludes iron NPs which appear to have no impact. There is considerable research that shows Ag NPs can alter the gut microbiome; however, there are also a few studies indicating there to be no impact. Furthermore, one study showed that Ag NPs can be formulated to alleviate colitis in mice. Several studies also demonstrated that TiO_2_ NPs have little impact on the gut microbiome while others suggested otherwise. Studies on ZnO NPs showed they can alter the gut microbial composition as well as their metabolic activity through measurements of SCFA production. Iron NPs appear to have no impact on the gut microbiota and SiO_2_ NPs cause only minor changes; however, in both cases, there is a lack of studies investigating microbial composition. Specifically, there is little data on the in-vivo effect of dietary levels of TiO_2_ on microbial composition in terms of phyla and genera [72].

Overall, only ZnO NPs were found to have positive impacts on the gut as the other inorganic NPs discussed showed some negative impact. Ag NPs reduced mucus secretion and negatively impacted the intestinal epithelial microvilli and tight junctions, leading to colitis-like symptoms in mice [6,45]. In stark contrast, other studies showed that Ag NPs do not damage the intestinal barrier and in fact reduced colon damage scores in mice [49,51]. SiO_2_ NPs led to the destruction of the intestinal epithelial layer and liver fibrosis [6,65]. However, other studies showed SiO_2_ NPs have no impact on the gut, deeming them safe for use in foods [66,67]. TiO_2_ NP treatment led to the irregular arrangement of epithelial cells, increased length of intestinal villi, intestinal inflammation, and preneoplastic lesions [59,68]. Fe_2_O_3_ NPs altered the amounts of goblet cells, caused irregular distribution of epithelial cells, and increased apoptosis, resulting in more intracellular space [69]. By contrast, another study declared that Fe_2_O_3_ NPs did not cause any histopathological lesions [70]. Finally, ZnO NPs increased tight junction protein expression, inhibited apoptosis in enterocytes, and did not alter jejunal villus height and crypt depth [53]. Similarly, in a study of green synthesized ZnO NPs, antioxidant and anti-inflammatory effects were observed [71]. It is important to note, however, that there is a lack of studies on the impact of ZnO NPs on the gut.

While this review is focused on microbiome-mediated impacts of NPs on health, NPs are likely negatively impacting GI health via interaction with the immune system. For instance, a study showed that TiO_2_ and SiO_2_ NPs caused upregulation of MHC-II, CD80, and CD86 on dendritic cells. They also activated IL-1b-secretion in wild-type (WT) but not Caspase-1- or NLRP3-deficient mice [73]. This means that silica NPs induced apoptosis and TiO_2_ NPs increased reactive oxygen species (ROS) production. Interaction with immune cells allows NPs to alter the secretion of inflammatory cytokines, potentially leading to inflammatory diseases of the gut. In another study, rats orally treated with food-grade TiO_2_ for 7 days, the rats showed decreased levels of T-helper interferon-gamma secretion and increased occurrence of Th1/Th17 inflammatory responses [68]. This means there is a higher risk of developing Th17-driven autoimmune diseases and colorectal cancer. This also applies to NPs that improve GI health. For instance, mRNA expression of inflammatory cytokines (IFN-γ, IL-1β, TNF-α, and NF-κB) were reduced in piglets treated with ZnO NPs [53]. Thus, ZnO NPs are capable of downregulating proinflammatory cytokines, thereby alleviating weaning induced inflammation in piglets [53]. Thus, NPs contribute to disease pathogenesis through interaction with the immune system as well. More research should be done to clarify the relationship between the impact of NPs on the gut microbiome and the impact of NPs on the immune system in relation to disease pathogenesis.

### 7.2. Comparison between NP-Induced and Disease-Associated Alterations of the Gut Microbiome

There is a high risk of *Clostridium difficile* infection in hospitalized patients, leading to morbidity and mortality [74]. One study found that clearance of this infection from the murine GIT depended more on the gut microbial structure (before any antibiotic treatment) than on adaptive immunity. Researchers were able to predict, with 76.9% accuracy, whether mice would continue to be colonized with the infection. Ninety percent of the top operational taxonomic units (OTUs) that contributed most to the authors’ classification belonged to the phylum Firmicutes. OTU 52 and OUT 93, belonging to the family Lachnospiraceae, ranked highest in discriminating between groups and was abundant in mice that later cleared the infection. These two alone could classify mice that clear infection with 66.6% accuracy. As shown in Table 2, it has been reported that NPs can affect the levels of Firmicutes phyla in the gut microbiome. Thus, there is a potential for NP-induced changes to the gut microbiome to influence an individual’s ability to clear infections. Another study assessed stool samples of rheumatoid arthritis patients receiving disease-modifying antirheumatic drugs (DMARDs) [75]. qPCR was used to evaluate the gut bacteria and revealed an increase in relative expression units (REU) of Bacteroidetes and *Prevotella* species and a decrease in the REU of *Clostridium leptum* compared to healthy controls. This reveals that the gut microbiota may play a role in the clinical response to DMARDs in RA patients. It would be interesting to determine whether NP-induced changes to the gut microbiome could potentially improve or reduce the efficacy of therapeutic drugs.

An individual’s immune system attacking self-tissues leads to the development of autoimmune diseases, which have a worldwide incidence of 3–5% [76]. It is known that environmental factors such as lifestyle and diet play a role in pathogenesis and various changes to the gut microbiome are linked with specific autoimmune diseases. For instance, IBD is associated with dysbiosis. Additionally, Crohn’s disease and ulcerative colitis can be characterized by the overgrowth of Proteobacteria. Thus, it is possible that NP-induced changes to the gut microbiome could promote disease pathogenesis. It is important to note that damage is not limited to the gut. For instance, the gut microbiota is implicated in Rheumatoid arthritis which is an autoimmune disease in which the joints are attacked by the immune system, causing inflammation and pain. Systemic lupus erythematosus is another autoimmune disease for which the gut microbiota is implicated with a lower F/B ratio and an abundance of specific genera like *Prevotella*. Sjogren’s syndrome (SS) is an autoimmune inflammatory disorder causing reduced saliva, tears, and pancreatic juice. SS patient fecal samples have a 50% reduction in the genus *Faecalibacterium,* showing that the microbiome is potentially involved in disease pathogenesis. Finally, systemic sclerosis is a complex disease associated with microbial alterations such as reductions in *Faecalibacterium* and *Clostridium* [76]. These diseases are all related to microbial alterations, which is why chronic exposure of the gut to NPs through food is concerning. Various inorganic NPs can alter the gut microbiome, so it is important to consider their potential contribution to diseases associated with those microbial changes.

Table 3 has been constructed to make associations between NP-induced alterations of the gut microbiome and diseases characterized by those precise alterations. Overall, chronic exposure to NPs may be placing individuals at risk of developing various diseases by altering their gut microbiome to resemble diseased states (summarized in Table 3). It is important to note, however, that the opposite may also be true in cases where NPs alter the microbiome away from disease-related states. For example, TiO_2_ NPs can increase Firmicutes and Bacteroidetes, making them potentially useful for alleviating IBD as it has been shown that IBD samples have reduced Firmicutes and Bacteroidetes phyla [60,77]. Additionally, Ag, ZnO, and TiO_2_ NPs all cause an increase in Bacteroidetes, making them potentially useful as well [45,52,60]. The problem is that such NPs are altering many other microbes, making it difficult to discern the overall impact on health. The findings in Table 3 simply indicate that NPs can change the gut microbiota in ways that mimic the changes observed in IBS, IBD, and celiac disease as well as other diseases outside of the GIT.

### 7.3. Impact of NP Characteristics and Experimental Design on Study Findings

Based on the studies presented in Table 2, it can be concluded that the response of the microbiome to NP exposure differs depending on many variables beyond the NP being analyzed. This may account for the variations in findings for studies testing the same NP. For instance, there are variations in the dose, size, and coating of the NPs used. Additionally, oral exposure to NPs includes the administration of NPs in water or in food. Thus, there may be differences in study findings due to the method of oral exposure used. It is difficult to draw a clear connection between the characteristics of the NPs used and the study’s findings due to many other variables that are also changing between studies (ex: sample, analysis, animal model, duration of exposure, etc.). Future studies should use systematic experiments that can be interpreted to account for the impact of these variables on study findings. For instance, many studies tested various shapes and sizes of NPs, different formulations of NPs (anatase vs. rutile, food-grade vs. industrial-grade), different coatings, etc. and found that these variables had some impact on results [47,59,60]. For example, the microbial recognition genes TLR2 (in male rats), TLR4 (in female rats), and NOD2 were downregulated depending on the dose of NPs given and the sex of the rats [45]. Unfortunately, the exact mechanisms for how these variables affect results are unknown. A potential explanation is that NPs can interact with intestinal contents and form stable protein coronas which alter their surface properties ultimately impacting their antimicrobial activity [48]. Variables like dose, size, shape, and coating can influence the type of interaction the NP will have with intestinal contents thereby altering its impact on the microbiota. For example, food grade NPs reduce the pH of the colon more compared to industrial grade NPs [60]. Such interaction between the NP and the environment may greatly influence the resulting impact of the NP on the microbiome.

Additionally, it is important to note that there may be differences in results due to the type of samples used to analyze the microbial community as well as their origin. For instance, studies have used fecal, ileal, cecal, and colonic samples to obtain microbiota for analysis. Additionally, some of the fecal samples used originated from human donors while others came from animal models like mice and rats. A study analyzing the microbiota of newly diagnosed CD patients and healthy control patients only found differences in bacterial population when using mucosal samples whereas fecal samples produced no results [101]. Thus, it is likely that bacteria residing in the mucosal layer are a better indication of gut health in relation to disease. Mucus thickness also varies down the intestine along with the amount of microbiota which may lead to varying results depending on the section being used for sampling [1]. Therefore, the sample used and its origin, can also greatly impact study findings on the NP impacts on the gut microbiome.

### 7.4. Current Methodological Limitations and Future Directions

When sequencing 16S rRNA to evaluate microbial communities, it is important to note that PCR-based methods can detect both live and dead cells rendering the goal of detecting NP effects on the gut microbial composition pointless [45]. Additionally, authors have mentioned that Ag NP exposure could potentially affect cultivation efficacy, preventing the researcher from deciphering whether the observed cell death is due to methodology or true Ag NP-dependent toxicity [48]. Finally, only a fraction of bacteria can be cultivated as up to 70% of them may be present but unable to be cultured [48]. Due to the differences between the oral consumption of NPs by humans compared to that of animals, research findings discussed may not be directly applicable to humans [6]. Although researchers have used mice models to assess microbial composition, the human and mouse gut microbiota are very similar only at the phylum level, not at the genera or species level [9]. Moreover, 85% of the 16S rRNA sequence of mouse microbiota represents genera that do not exist in humans [81]. This makes findings difficult to interpret as most of the studies discussed measured microbial alterations through 16S rRNA sequencing (Table 2). There are studies, however, which used human samples to inoculate gut models, making them more representative of humans. It is important to note that other methods than sequencing and cultivation exist for assessing microbial composition. For instance, some of the studies analyzed functional aspects like metabolites (e.g., SCFA) and gas production to evaluate the gut microbiome [46,50,54,62].

The varied methods of NP exposure are another potential explanation for conflicting study results. For example, some researchers prefer voluntary oral intake as it represents human exposure more closely [9]. However, research has demonstrated that the aging of food pellets affects NP toxicity. This was shown by assessing pellets 4 and 8 months after Ag NP incorporation. In the 4-month-old condition, Ag NPs significantly altered gut microbiota but the increase in F/B seen in fresh pellet conditions became insignificant. In the 8-month-old condition, there was no effect of Ag NPs on gut microbiota and the F/B balance was unaltered. Because Ag+ ion release is thought to be the major mechanism of Ag NP toxicity in bacteria, authors hypothesized there may be alterations in Ag NP form over time that could explain the differences in toxicity. Indeed, newly acquired Ag NP pellets release Ag+ more rapidly compared to aged pellets. Authors also found that aged pellet NPs underwent sulfidation which may be the reason for reduced Ag NP toxicity. It was noted that new and old NPs conserved in air, as opposed to food pellets, did not show differences in Ag+ release over 21 days. Taken together, this indicates future studies should not use food pellets to administer NPs. Moreover, if they chose to, sulfidation states should be documented to aid in the interpretation of findings. Given these findings, it may be possible to reduce food NP toxicity by managing how aged it is at the point of consumption.

Considering these limitations, future studies should investigate the inoculation of the human gut microbiota into gnotobiotic mice to increase the applicability of findings to humans. More long-term research should also be done to better resemble the chronic state of human exposure [59]. More experiments should be conducted to determine the precise impact of confounding variables (NP size, dose, method of administration, animal model, etc.) on study findings to explain the currently conflicting results. Finally, studies should investigate the potential for the gut microbiota to recover from dysbiosis, bringing insight into the permanence of NP-induced damage. Only one of the many studies discussed has incorporated this into their study finding that microbial composition was restored after treatment cessation [58].

### 7.5. Potential Systematic Use of Probiotics and NPs as Therapeutic Agents against Gut Dysbiosis

Given that NPs can negatively alter the gut microbiota in precise ways, it may be possible to formulate probiotics that can potentially reverse the negative effects of NPs. For instance, if NPs tend to create pro-inflammatory states in the gut, probiotics known to have anti-inflammatory effects may be used as therapeutics. In the study using in-vitro batch fermentation models inoculated with human fecal matter, interactions between 1 ug/mL Ag NPs, the intestinal microbiota, and the probiotic Bacillus subtilis were assessed [50]. The F/B ratio increased with Ag NPs but cocultures with the probiotic had the lowest F/B ratio. Similarly, the probiotic was able to ameliorate Ag NP induced functional differences in cell motility, translation, transport, and xenobiotics degradation. In this way, probiotics should be considered as potential therapeutics for NP-induced damage to the gut.

Once NPs are fully characterized based on their physicochemical properties and can be predicted to cause precise changes to the gut microbiome, it may be possible to use them to correct dysbiosis associated diseases. For instance, data has shown that normalizing the number of *E. coli* can promote remission in patients with ulcerative colitis [51]. In the study that tested two aqueous suspensions of Ag NPs on mouse models of ulcerative colitis and Crohn’s disease, NPs were found to have anti-inflammatory activity as they improved colitis [51]. It was noted that this effect was related to the shape and diameter of the NP as Ag NP1 (294 nm, spherical) had a weaker effect compared to Ag NP2 (122 nm, irregular shape). Thus, the smaller diameter and more irregularly shaped NPs contribute more to improving colitis. Since both Ag NPs alleviated colitis, they may be potential therapeutic agents for IBD [51]. While many studies conflict with these results, finding the precise reasons for such differences in findings (i.e., physicochemical properties of NPs, host environment, etc.) may lead to the production of NPs that can have a positive impact on health instead of negative ones. This would essentially allow for various industries to modify the kinds of NPs they utilize to reduce their negative impact on health.

## 8. Conclusions

NPs have been widely used in various industries from food to cosmetics. However, research suggests they may negatively impact human health. Based on the studies reviewed in this article, all the discussed inorganic NPs can alter the composition of the gut microbiota except for iron NPs. Inorganic NPs can also negatively impact the GIT; however, there are also opposing studies which suggest otherwise. NP-induced alterations to the gut microbiota and intestinal barrier have the potential to lead to various inflammatory diseases. Further systematic research must be done to fully determine the impact of chronic exposure to NPs with consideration for methodological variables that may be contributing to conflicting findings. Further research must also be done on the combinatorial nature of the exposure, considering humans are exposed to various NPs at once. Finally, the mechanism of action of NP-induced disruption of the gut microbiota and intestinal tract must be further elucidated.

## Figures and Tables

**Table 1:** Applications of inorganic nanoparticles in various industries.

**NP.**	**Optical**	**Electronic**	**Biomedical**	**Textile**	**Food [5]**
Ag [6]	Light-harvesting applications. Solar panels. optical enzyme biosensing. Enhance semiconductor efficiency.	A conductive filler in electronically conductive adhesives (ECAs) for reducing electrical loss. Micro packaging systems in electrical devices.	Therapeutics, Imaging, Diagnostics.	Deposited in fabrics for antibacterial and anti-odor properties. Waterproof textile materials. Tap water purification devices. Deposited on zeolite, sand, fiberglass, resin substrates, and used in groundwater purification.	Antimicrobial agents in food packaging materials. In food additive E174, used in surface coatings for sweets [12].
**NP**	**Cosmetics**	**Agriculture**	**Biomedical**	**Textile/Rubber**	**Food**
ZnO	Chemical industry catalyst for cosmetic products [11].	Used in food crops to increase yield [13]. Colloidal solution of ZnO NPs is in fertilizers [13]. Used as pesticides [13].	Potential use in anticancer drug delivery, diabetes treatments, anti-inflammatory activity, bioimaging and pathology [14]. Urea, cholesterol, H_2_O_2_, phenol, and glucose biosensors [15].	Acids vulcanization in rubber for tire manufacturing [16]. Cement [16]. Clear varnishes for wood and furniture [16]. Plastic glasses [16].	Source of zinc in supplements [5]. Antimicrobial agent or UV light absorber in food packaging [10,11].
**NP**	**Construction**	**Agriculture**	**Biomedical**		**Food**
SiO_2_	Paints, coats tiles, concrete, cement, pipes, glasses, solutions, coats [17].	Controlled release of commercial pesticides [18]. Delivery vectors for fertilizers [18]. Optical sensory for melamine, imaging of copper ions in tap water [18].	Mesoporous silica NPs (MSNPs) are used to detect hydrogen peroxide and deliver controlled drug release in heart failure [19]. Employed catalysis [20,21]. Energy storage [22,23]. Drug carrier for ophthalmological and osteoporotic diseases and diabetes [24].		Used in additive E551 as anticaking agent for powdered foods (i.e., salts, icing, sugar, spices, dried milk, dry mixes) [5].
**NP**	**Construction**	**Magnetic**	**Biomedical**		**Food**
Fe_2_O_3_	Iron oxide pigment is used in coloring concrete, brick, and tile [25].	Magnetic recording media for coercivity [26]. Soft magnetic materials (ex: Nanocrystalline iron alloys with phase-separated magnetic grains) [26].	Contrast agent for MRI [27]. Drug carrier for targeted drug delivery [27]. Gene therapy [27]. Therapeutic agents based on hyperthermia [27]. Nano adjuvant for vaccine or antibody production [27].		Enzyme immobilization [28]. Protein separation [28]. Food analysis [28]. Protein purification [28]. Colorant [5]. Source of bioavailable iron [5]. Mineral-fortified supplements [5].
**NP**	**Construction**	**Agriculture**	**Biomedicine/Cosmetics**	**Technology**	**Food**
TiO_2_	Surface coatings to increase adherence, firmness, anti-scratch, self-cleaning [17].	Soil amendment or foliar spray to enhance crops, photosynthetic rate, and immunity. Wastewater treatment [29].	Nanotherapeutics like photodynamic therapy (PDT) and articulating prosthetic implants. [30]. Sunscreen and hyperpigmentation treatments [30].	Semiconductors for dye-sensitized solar cells [30]. Photocatalytic coating materials for self-cleaning buildings. Anti-fog car mirrors [30]. Air purifying titanium mesh filter [30]. In photodegradation of toxic dyes and pharmaceutical drugs [30].	Used in E171 food colorants [6]. Has optical properties that lighten various foods [11,30].

**Table 2:** Summary of studies measuring nanoparticle (NP) effects on the gut microbiota based on NP characteristics, experimental design, analytical techniques, and findings.

NP	NP Characteristics (Dose, Size, Coating, Shape)	Experimental Design (Model, Administration, Duration, Sampling)	Measurement Technique	Findings
**Ag** [45]	9, 18, 36 mg/kg bw/day; 10, 75, 110 nm; Citrate-stabilized.	Sprague-Dawley rats. Oral gavage twice daily for 13 weeks. Cultured ileal tissues (*n* = 5).	Quantitative PCR	Decreased Firmicutes. Increased Bacteroidetes. Decreased *Lactobacillus.* Increased *Bifidobacterium.*
**Ag** [6]	2.5 mg/kg bw/day; 12 nm.	CD-1 (ICR) male mice. Oral gavage for 7 days. Used fecal samples.	16S rRNA pyrosequencing	Decreased F/B ratio. Alistipes, *Bacteroides*, and *Prevotella* increased. *Lactobacillus* decreased.
**Ag** [47]	3.6 mg/kg bw/day; 45 nm (cube) and 50 nm (sphere); PVP-coated; cube and sphere shaped.	Male Sprague Dawley rats. Oral exposure lasting 14 days. Analyzed fecal samples.	16S rRNA sequencing	Cube-shaped Ag NPs Reduced *Clostridium* spp., *Bacteroides uniformis*, *Christensenellaceae*, and *Coprococcus eutactus.* Sphere-shaped Ag NPs reduced *Oscillospira* spp., *Dehalobacterium* spp., *Peptococcaeceae, Corynebacterium* spp., and *Aggregatibacter pneumotropica.*
**Ag** [9]	11.4, 114, 1140 ug/kg bw/day; 55 nm; PVP-coated.	C57BL/6 female mice. 28 days treatment with food pellets supplemented with NPs. Analyzed fecal microbiota.	16S rRNA sequencing	F/B ratio increased with dose. *Coprococcus*, *Lactobacillus*, and *Blautia* increased*. Bacteroides* and *Mucispirillum* decreased.
**Ag** [48]	10 mg/kg bw/day; 20 nm and 110 nm; PVP or citrate coated.	Male C57BL/6NCrl mice. Oral gavage daily for 28 days. Cecal samples used.	16S rRNA sequencing (V3–V5 hypervariable region)	No changes in phylum composition, microbial community structure, or diversity.
**Ag** [58]	100 mg/day; 30–50 nm.	Human Gut Simulator system (HGS) seeded with human distal gut microbiota (3 males with no use of antibiotics or probiotics within 6 months). Treated for 7 days, followed by 7 days without NP treatment.	16S rRNA sequencing (V4 region)	Microbial population density decreased drastically. Microbiota was restored upon treatment cessation.
**Ag** [50]	1 mg/mL; 14 nm; capped with Sodium citrate.	In-vitro batch fermentation model inoculated with human fecal matter (4 healthy individuals who did not take probiotics more than 1 month before sampling).	16S rRNA sequencing (V3–V4 region). Identified key taxa using Fluorescent in-situ hybridization.	Core bacterial community was unchanged. Amount of rare species drastically changed. F/B ratio increased. Levels of *Faecalibacterium prausnitzii* and *Clostridium coccoides*/*Eubacterium rectales* taxa were negatively altered. Caco-2 cell monolayers were unaffected.
**ZnO** [52]	25, 50, 100 mg/kg,; ~30 nm.	Hens were fed NPs for 9 weeks. Sampled ileal microbiota.	16S rRNA sequencing (V3–V4 region)	Dose dependently reduced bacterial community richness, decreased Firmicutes and *Lactobacillus*, increased Bacteroidetes, *Fusobacteria* and Bacilli.
**ZnO** [53]	600, 2000 mg Zn/kg; 23 nm.	Crossbred weaning piglets. Treated for 14 days with ZnO NP supplemented basal diets. ileal, cecal, and colonic samples used.	16S rRNA sequencing (V3–V4 region)	Bacterial richness and diversity increased in the ileum but decreased in the cecum and colon. Increased *Streptococcus* and decreased *Lactobacillus* in the ileum. Increased *Lactobacillus* and decreased *Oscillospira* and *Prevotella* in the colon.
**ZnO** [54]	0.01 ug/L; 10 nm.	Model colon reactor. Two 5- day long experiments. Human microbial sample (26-year-old female with no use of antibiotics in over 8 months).	Phenotypic Analysis of extracellular polymeric substance, surface charge, hydrophobicity, cell concentration, SCFA production.	Hydrophobicity increased, sugar content of the extracellular polymeric substance became more negative, conductivity decreased, and the cell’s radius decreased. SCFA production was unchanged.
**TiO_2_** [60]	252–864 nm (industrial grade), 212–315 nm (food grade). Coated with inorganic phosphate.	Bench-scale model colon reactor. Exposures spanned 5 days. Used human fecal material from the colon (healthy, 26-year-old female free of antibiotics for 8 months).	Phenotypic characterization: bacterial tag-encoded pyrosequencing (28F-388R primer). Assigned operational taxonomic units.	Industrial grade: Reduced Proteobacteria by 67%. Firmicutes and Bacteroidetes increased Food grade: Decreased Proteobacteria by 13%. Minor increase in Firmicutes and Bacteroidetes.
**TiO_2_** [57]	100, 250 ppm; 25 nm; E171-1 and E171-6a food-grade formulations used.	Chemostat bioreactor, inoculated with a defined model intestinal bacterial community (MET-1). Food-grade TiO_2_ NP exposure for 48 h.	PCR-amplification followed by 454 pyrosequencing and phylogenetic distributions.	Decreased *Bacteroides ovatus*, increased *Clostridium cocleatum*. No major effect on gut microbiota.
**TiO_2_** [6]	2.5 mg/kg bw/day; 16 nm.	Male CD-1 (ICR) mice. Oral gavage for 7 days. Used fecal samples.	16S rRNA Pyrosequencing	Microbial composition and GIT histology was unchanged.
**TiO_2_** [59]	100 mg/kg per day; 15.9 nm (rutile); 20.1 nm (anatase).	Male C57BL/6 mice. Oral administration of the two crystalline phases via gavage for 28 days. Extracted fecal samples.	16S rRNA pyrosequencing	Rutile form: Increased Proteobacteria and *Rhodococcus.* Elongated intestinal villi and caused irregular arrangement of gut epithelial cells. Anatase form: Increased *Bacteroides*. Both forms caused a decrease in *Prevotella*.
**TiO_2_** [62]	0, 2, 10, 50 mg/kg; 29 nm; Spherical anatase crystals.	Sprague-Dawley rats. Administration was via oral gavage daily for 90 consecutive days. Samples used were rat feces.	16S rRNA sequencing (V3-V5 region)	Hepatotoxicity observed at the highest dose. Increase in *Lactobacillus reuteri* and decrease in *Romboutsia*.
**TiO_2_** [58]	100 mg/day; 25 nm.	HGS system used. 7 days of NP administration plus 7 days of no treatment. Used distal gut microbiota samples (3 males 27–31 years old with no use of antibiotics or probiotics within 6 months).	16S rRNA sequencing (V4 region)	Did not reduce microbial population density drastically. Microbial community was restored upon treatment cessation.

F/B: Firmicutes/Bacteroidetes.

**Table 3:** Comparison between NP-induced and disease-associated alterations of the gut microbiome.

NP(s)	Microbial Alteration due to NP Exposure	Association of Microbial Alteration with Disease
Ag	13–73% reduction in *Faecalibacterium prausnitzii* [46].	*Faecalibacterium* promotes immune tolerance. Thus, its reduction is linked with immune dysfunction and recurrence of Crohn’s disease [3,78]. Its reduction is also the most prominent feature of IBD [79,80]. *Faecalibacterium prausnitzii* is also lower in patients with celiac disease compared to healthy individuals [43].
Ag	Increased F/B ratio [45].	Increased Firmicutes compared to Bacteroidetes is associated with higher energy reabsorption and obesity [81,82]. Reduction in Bacteroidetes is related to rheumatoid arthritis [83]. IBS patient fecal samples showed increased Firmicutes- and decreased Bacteroidetes-related taxa [84].
Ag	Decreased *Alistipes* [6].	*Alistipes finegoldii* reduction is linked with Sjögren’s syndrome [85].
Ag	Increased *Bifidobacterium* [45].	An increase in *Bifidobacterium* is associated with systemic sclerosis [86].
Ag	Reduced *Clostridium* spp. [47].	Clostridia-like bacterium are reduced in systemic sclerosis [86]. *Clostridium coccoides* reduction is linked to rheumatoid arthritis [87]. Reduction in Firmicutes, specifically *Clostridium*, is related to IBD [79].
Ag	Cube-shaped NPs Reduced *Christensenellaceae* [47].	Reduced *Christensenellaceae* is linked to systemic lupus erythematosus [88,89].
TiO_2_	Increased *Clostridium cocleatum* [57].	An increase in clostridia-like bacteria is linked with rheumatoid arthritis [90].
TiO_2_	Increased Firmicutes and Bacteroidetes [60].	An increase in Firmicutes is linked with Rheumatoid arthritis and Sjogren’s syndrome [83,91]. Infants with high genetic risk for celiac disease have increased proportions of Firmicutes [44]. *Bacteroides* are significantly higher in celiac disease [92].
ZnO	Reduced microbiome diversity [53].	Infant gut microbiome diversity is reduced in those who develop allergy, asthma, or malnourishment [3]. Reduced diversity is related to old-age frailty [3]. IBD patients have decreased microbial diversity and complexity [76,77]. Children with severe ulcerative colitis have reduced microbiome richness and diversity [80].
ZnO	Decreased Firmicutes [52].	Decrease in Firmicutes is found in systemic lupus erythematosus [88]. IBD patients show a decrease in Firmicutes [77]. Firmicutes were less abundant in celiac disease compared to controls [93].
Ag ZnO	Ag NPs decreased *Lactobacillus* [6,45]. ZnO NPs decreased *Lactobacillus* [52]. ZnO NPs decreased *Lactobacillus* in the ileum [53].	Reduced *Lactobacillaceae* is related to systemic lupus erythematosus [94,95]. Lactobacillus is significantly reduced in active celiac disease [96].
Ag ZnO TiO_2_	Increased Bacteroidetes [45,52,60].	Bacteroidetes are increased in the guts of individuals with Sjögren’s syndrome [97].
TiO_2_ Ag ZnO	TiO_2_ NPs increased *Lactobacillus reuteri* [62]. Ag NPs Increased *Lactobacillus* [9]. ZnO NPs increased *lactobacillus* in the colon [53]. TiO_2_ NPs Increased *Lactobacillus reuteri* [62].	*Lactobacillaceae* are increased in people with rheumatoid arthritis and systemic sclerosis [95,98].
SiO_2_ TiO_2_	SiO_2_ NPs increased Proteobacteria [6]. Rutile form of TiO_2_ NPs increased Proteobacteria [59].	Proteobacteria are increased in IBD [77,76]. Infants with high genetic risk of celiac disease have increased proportions of Proteobacteria [44,99].
Ag TiO_2_	Ag and TiO_2_ NPs increased *Bacteroides* [6,59].	*Bacteroides* are significantly more abundant in celiac disease patient stool and biopsy samples [96].
Ag	Ag NPs increased *Prevotella* [6].	*Prevotella* is higher in patients with celiac disease compared to controls [43].
Ag TiO_2_	Ag NPs reduced *Bacteroides ovatus* [46]. TiO_2_ NPs caused minor reductions in *Bacteroides ovatus* [57].	Active celiac disease patients have lower abundance of *Bacteroides ovatus* compared to controls [100].

F/B: Firmicutes/Bacteroidetes

## Abbreviations

CD	Crohn’s disease
CDK	Cyclin-dependent kinase
CRC	Colorectal cancer
GIT	Gastrointestinal tract
IBD	Inflammatory bowel disease
IBS	Irritable bowel syndrome
NP	Nanoparticle
SiO_2_	Silicon dioxide, silica
TiO_2_	Titanium dioxide
TLR	Toll-like receptors
UC	Ulcerative colitis
ZnO	Zinc oxide
*FOXP3*	Forkhead box P3 gene
*GPR43*	G-protein-coupled receptor 43 gene
*IL*	Interleukin gene
*TGF-β*	Transforming growth factor β gene
*NOD2*	Nucleotide-binding oligomerization domain-containing protein 2 gene
*IFN-γ*	Interferon gamma gene
*TNF-α*	Tumor Necrosis Factor Alpha gene
*NF-κB*	Nuclear factor kappa B gene
